# The Impact of Sleep Deprivation in the Treatment of Depression: A Literature Review

**DOI:** 10.1192/j.eurpsy.2024.808

**Published:** 2024-08-27

**Authors:** J. Camilo

**Affiliations:** Psychiatry, CHTMAD, Vila Real, Portugal

## Abstract

**Introduction:**

Depression is a pervasive and debilitating mental health disorder that affects millions of individuals worldwide. Despite the availability of various treatment modalities, a significant proportion of patients continue to experience inadequate symptom relief and persistent emotional suffering. Sleep disturbance is a common symptom of depression, and emerging evidence suggests that manipulating sleep patterns through sleep deprivation may hold potential therapeutic benefits. This literature review aims to explore the role of sleep deprivation as an adjunctive treatment for depression, shedding light on its mechanisms and potential outcomes.

**Objectives:**

To investigate the historical context and theoretical underpinnings of sleep deprivation in depression treatment; to examine the methods and protocols used in studies involving sleep deprivation as a therapeutic intervention for depression; to analyze the empirical evidence regarding the efficacy of sleep deprivation in ameliorating depressive symptoms; to assess the safety and feasibility of implementing sleep deprivation in clinical practice; to discuss the potential mechanisms underlying the antidepressant effects of sleep deprivation.

**Methods:**

A systematic review of the literature was conducted.

**Results:**

The review identified a diverse body of literature exploring the potential benefits of sleep deprivation in depression treatment. Preliminary findings suggest that acute sleep deprivation may lead to rapid and transient improvements in mood among individuals with depression. Various protocols, including total and partial sleep deprivation, have been investigated, demonstrating differential effects on depressive symptoms. Additionally, potential mechanisms underlying these effects, such as alterations in neurochemical pathways and circadian rhythms, have been proposed.

**Conclusions:**

Sleep deprivation as an adjunctive treatment for depression is a promising but complex intervention that requires further investigation. While some studies have reported significant improvements in mood following sleep deprivation, the sustainability of these effects and the potential long-term consequences remain uncertain. Moreover, the optimal protocols, safety guidelines, and patient selection criteria need to be established for clinical application. Future research should focus on elucidating the mechanisms involved and conducting well-designed randomized controlled trials to determine the efficacy and safety of sleep deprivation in the context of depression treatment. This review underscores the importance of considering sleep as a modifiable factor in the comprehensive management of depression.

**Disclosure of Interest:**

None Declared

